# IS AGING AN ACQUIRED MITOCHONDRIAL DISEASE?

**DOI:** 10.1093/geroni/igz038.1458

**Published:** 2019-11-08

**Authors:** Alessandro Bitto

**Affiliations:** University of Washington, Seattle, Washington, United States

## Abstract

Mitochondrial dysfunction is a hallmark of aging, but severe mitochondrial dysfunction leads to rare childhood disorders such as Leigh Syndrome. This session explores the similarities and differences between normative aging and mitochondrial disease and the potential for interventions to positively impact both conditions.

